# Qualitative and Quantitative Differences in Herbivore-Induced Plant Volatile Blends from Tomato Plants Infested by Either *Tuta absoluta* or *Bemisia tabaci*

**DOI:** 10.1007/s10886-016-0807-7

**Published:** 2017-01-03

**Authors:** Diego B. Silva, Berhane T. Weldegergis, Joop J.A. Van Loon, Vanda H. P. Bueno

**Affiliations:** 1 Laboratory of Biological Control, Department of Entomology, Federal University of Lavras, P.O.Box 3037, Lavras/MG, 37200-000 Brazil; 2 Laboratory of Entomology, Wageningen University, P.O. Box 16, 6700 AA Wageningen, The Netherlands

**Keywords:** Tomato, *Tuta absoluta*, *Bemisia tabaci*, HIPVs, GC-MS

## Abstract

Plants release a variety of volatile organic compounds that play multiple roles in the interactions with other plants and animals. Natural enemies of plant-feeding insects use these volatiles as cues to find their prey or host. Here, we report differences between the volatile blends of tomato plants infested with the whitefly *Bemisia tabaci* or the tomato borer *Tuta absoluta.* We compared the volatile emission of: (1) clean tomato plants; (2) tomato plants infested with *T. absoluta* larvae; and (3) tomato plants infested with *B. tabaci* adults, nymphs, and eggs. A total of 80 volatiles were recorded of which 10 occurred consistently only in the headspace of *T. absoluta*-infested plants. Many of the compounds detected in the headspace of the two herbivory treatments were emitted at different rates. Plants damaged by *T. absoluta* emitted at least 10 times higher levels of many compounds compared to plants damaged by *B. tabaci* and intact plants. The multivariate separation of *T. absoluta*-infested plants from those infested with *B. tabaci* was due largely to the chorismate-derived compounds as well as volatile metabolites of C_18_-fatty acids and branched chain amino acids that had higher emission rates from *T. absoluta*-infested plants, whereas the cyclic sesquiterpenes α- and β-copaene, valencene, and aristolochene were emitted at significantly higher levels from *B. tabaci*-infested plants. Our findings imply that feeding by *T. absoluta* and *B. tabaci* induced emission of volatile blends that differ quantitatively and qualitatively, providing a chemical basis for the recently documented behavioral discrimination by two generalist predatory mirid species, natural enemies of *T. absoluta* and *B. tabaci* employed in biological control.

**Electronic supplementary material:**

The online version of this article (doi:10.1007/s10886-016-0807-7) contains supplementary material, which is available to authorized users.

## Introduction

The defense of plants against insect herbivores involves different strategies. Plants can defend themselves directly through the production of morphological structures on the leaf surface e.g., trichomes and by producing toxic compounds that deleteriously affect the behavior or development of the herbivores (Schoonhoven et al. 2005). Plant defense also can involve indirect mechanisms, including the production and release of volatile organic compounds (VOCs) as a response to herbivore feeding, commonly known as herbivore-induced plant volatiles (HIPVs) that provide important foraging cues for natural enemies of the herbivores (Dicke et al. 2009; Turlings et al. 1990).

Herbivore-induced plant volatiles can be comprised of hundreds of compounds (Dudareva et al. 2006), varying quantitatively and qualitatively depending on both abiotic and biotic factors, and are specific to each plant – herbivore association (Benelli et al. 2013; Ingegno et al. 2011). When a plant is attacked by a leaf-chewer or by a phloem feeder or when attacked by more than one organism, it reacts differently (Dicke et al. 2009; Gosset et al. 2009; Zhang et al. 2009, 2013). For instance, chewing insects, such as caterpillars, predominantly activate the jasmonic acid (JA)-mediated signaling pathway, whereas feeding by phloem-sucking herbivores predominantly activates the salicylic acid (SA) signaling pathway (Walling 2000), each resulting in the synthesis of specific blends of HIPVs that attract natural enemies of herbivorous arthropods (Heil 2014; Wei et al. 2014; Zhang et al. 2013).

Tomato (*Solanum lycopersicon* L.) is an important fruit crop with high susceptibility to insect herbivory. It is a host plant for two important pests worldwide, belonging to two different feeding guilds, the tomato borer, *Tuta absoluta* (Meyrick) (Lepidoptera: Gelechiidae), and the phloem-sucking whitefly *Bemisia tabaci* (Gennadius) (Hemiptera: Aleyrodidae). In the absence of any measure of control, infestation by these insect herbivores can result in up to 100% production loss (Desneux et al. 2010; Navas-Castillo et al. 2011). To reduce economic damage to tomato cultivation, insecticides are commonly applied (Zalom 2003). The large scale use of insecticides causes environmental concerns and is harmful for natural enemies. Therefore, efficient sustainable pest management strategies are needed. Being an annual plant with a short life cycle, tomato would benefit from recruiting natural enemies even more than perennial plants (Hilker and Meiners 2006). For the development of effective and successful pest control strategies, it is important to elucidate the chemical ecology of tritrophic systems of natural enemies, herbivores, and host plants. Identified semiochemicals can be used to manipulate the abundance and distribution of natural enemies (Hilker and Fatouros 2015).

Herbivore-induced plant volatile blends released by tomato plants in response to herbivore infestation attract carnivorous natural enemies such as predators and parasitoids (Abbas et al. 2014; Moayeri et al. 2007a; Rodriguez-Saona et al. 2005). HIPV blends produced in response to chewing and phloem-sucking herbivores increase the attraction of mirid predators (De Backer et al. 2015; Moayeri et al. 2007b; Pérez-Hedo et al. 2015). Differences in HIPV blend composition enable carnivores to make choices among available plant-herbivore combinations.

It was shown recently that the mirid predators *Macrolophus pygmaeus* Rambour and *Nesidiocoris tenuis* (Reuter) (both Hemiptera: Miridae) preferred the HIPV blends of tomato plants infested with *B. tabaci* or *T. absoluta* over the volatile blend emitted by uninfested tomato plants (Lins et al. 2014). In the current study, we aimed to identify differences in HIPV blends from tomato plants infested with whitefly *B. tabaci* or the tomato borer *T. absoluta*, which may allow the predators to discriminate among the herbivore-infested and uninfested tomato plants.

## Material and Methods

### Plants and Insects

Tomato plants *Solanum lycopersicon* L. cv. Moneymaker were grown in a greenhouse compartment (25 ± 2 °C, 70% ± 10% R.H., L16:D8). Plants of 30–35-d-old (5–6 leaves and 20–25 cm in height) were used in the experiments.

Adult *T. absoluta* were kept in mesh cages (60 × 40 × 40 cm) with a potted tomato plant in a controlled room (25 ± 2 °C, 60 ± 10% R.H., L16:D8) to allow oviposition until larvae hatched; uninfested tomato leaves were introduced into the cages when necessary to ensure *ad libitum* feeding.

*Bemisia tabaci* was reared under the same greenhouse conditions, however, in another compartment. Adults were kept in mesh cages on potted tomato plants. Once per week a new cohort of adults was started on uninfested plants.

Nymphs and adults of *M. pygmaeus* and *N. tenuis* were supplied by Koppert Biosystems (Berkel en Rodenrijs, The Netherlands and Almeria, Spain, respectively), kept in climate cabinets (25 ± 1 °C, 70 ± 5% R.H., L16:D8) in cages (60 × 40 × 40 cm) containing a potted tomato plant. Eggs of *Ephestia kuehniella* Zeller (Lepidoptera: Pyralidae) were offered ad libitum every 3 d as food.

### Plant Treatments

To characterize the differences in plant volatiles released in response to attack by *T. absoluta* and *B. tabaci*, we collected headspace volatiles of tomato plants subjected to different herbivore treatments. All tomato plants for the experiment were treated in a controlled room (25 ± 2 °C, 70% R.H., L16:D8). Plants were subjected to three treatments: (1) control, i.e., without herbivory, (2) *T. absoluta* infestation, (3) *B. tabaci* infestation.

Herbivore-infested and control plants were kept in separate mesh cages (60 × 40 × 40 cm) and in separate climate-controlled rooms (25 ± 2 °C, 60 ± 10% R.H., L16:D8).

Tomato plants, 30–35-d-old were covered with organza bags, and five couples of *T. absoluta* of up to 3-d-old were released into each bag. Females were allowed to lay eggs for 48 h, and then the adults were removed. According to Silva et al. (2015), five *T. absoluta* females lay 125 eggs/day; the egg survival at 25 °C is 98%, resulting in an estimated 245 first instar larvae hatching after 4–5 d. Larvae were allowed to feed for 72 h (Lins et al. 2014).

Fifty adults of *B. tabaci* were released in a cage (60 × 40 × 40 cm) with tomato plants. Ten days after infestation, the plants with adults, eggs, and nymphs were used in the tests (Lins et al. 2014).

### Headspace Collection of Plant Volatiles

Prior to volatile collection, pots in which the plants were growing were carefully wrapped with aluminum foil. The plant sample was placed in a 30 L glass jar and was left for 30 min for acclimatization prior to volatile collection. Subsequently, a stream of charcoal filtered air was passed over the plant for 2 h at a flow rate of 200 ml min^−1^, and volatiles were collected by passing the air stream through a stainless steel cartridge filled with 200 mg Tenax TA (20/35 mesh; CAMSCO, Houston, TX, USA) (Weldegergis et al. 2015). Immediately after the collection of volatiles, plant shoot fresh weight was measured, and the Tenax TA cartridges with volatiles were dry-purged for 15 min under a stream of nitrogen (N_2_, 50 ml min^−1^) at room temperature (21 ± 2 °C) to remove moisture, and then stored at ambient temperature until analysis. For each treatment, 10 replicate plants were sampled. In order to correct for any non-plant volatile contribution, volatiles were collected from aluminum wrapped pots filled with soil only.

### Analysis of Plant Volatiles

Headspace samples were analyzed with a Thermo Trace Ultra gas chromatograph (GC) coupled to a Thermo Trace DSQ quadrupole mass spectrometer (MS), both from Thermo Fisher Scientific (Waltham, MA, USA) using a protocol described by Cusumano et al. (2015). The collected volatiles were released from the Tenax TA thermally on Ultra 50:50 thermal desorption unit (Markes, Llantrisant, UK) at 250 °C for 10 min under a helium flow of 20 ml min^−1^, while re-collecting the volatiles at 0 °C on an electronically cooled sorbent trap (Unity, Markes). The volatiles were transferred in splitless mode to the analytical column (ZB-5MSi, 30-m × 0.25-mm I.D. × 0.25-μm film thickness with a 5-m built-in guard column; Phenomenex, Torrence, CA, USA) placed in the GC oven. Further separation was achieved by ballistic heating of the cold trap to 280 °C, where it was kept for 10 min. The GC oven temperature was initially held at 40 °C for 2 min and then raised at 6 °C min^−1^ to a final temperature of 280 °C, which was maintained for 4 min under a column flow of 1 ml min^−1^ in constant flow mode. At 70 eV EI-mass spectra were acquired while scanning from *m*/*z* 35 to 400 at a rate of 4.70 scans s^−1^. The MS transfer line and ion source were set to 275 and 250 °C, respectively. Tentative identification of compounds was based on comparison of mass spectra with those reported in the NIST 2008 MS library. Experimentally calculated linear retention indices (LRI) were also used as an additional criterion to identify target compounds. We quantified the importance of each VOC in the separation between treatment groups by using Partial Least Squares - Discriminant Analysis (PLS-DA) (Barker and Rayens 2003). Relative quantification of peak areas of individual compounds was done using the integrated absolute signal of a quantifier ion in the selected ion monitoring (SIM) mode. The individual peak areas of each compound were computed into peak area per gram shoot biomass to correct for differences in size of individual plants and were further used in the statistical analysis. Volatiles from the compressed air, glass jars, pots, and soils as well as cleaned Tenax TA adsorbents and the analytical system itself were treated as blank samples and used to correct for artefacts during analysis.

### Data Analysis

Prior to analysis, the raw data of corrected peak areas were tested for normality and homogeneity of variances using the *Shapiro-Wilk* and *Bartlett* tests, respectively. To test for significant differences among treatments, the non-parametric *Kruskal-Wallis* test was used since their distribution did not meet the assumptions for standard parametric ANOVA. Statistical analyses were performed using R statistical software (R Core Team 2014). For volatile emission patterns, the corrected peak areas divided by plant shoot fresh weight were log-transformed, mean-centered, and scaled to unit variance prior to analysis using a multivariate data analysis approach: projection to latent structures discriminant analysis (PLS-DA) using SIMCAP + 12.0 software (Umetrics AB, Umeå, Sweden). PLS-DA is a method commonly used for pattern recognition and group separation among samples of different treatments based on available qualitative and quantitative information (Wold et al. 2001). PLS-DA provides score plots displaying visually recognized sample structure separating treatment groups according to model components, and complementary loading plots, displaying the contribution of each variable (in this case volatile compound) to these components separating the treatment groups as well as the relationships among the variables themselves.

## Results

Among headspace volatiles released by tomato plants exposed to herbivory by *T. absoluta* (TA), *B. tabaci* (BT), or no herbivory (control, C), a total of 80 VOCs were assigned, of which 68 compounds were present in all treatments, whereas 75 compounds were detected in at least one of the herbivory treatments (Table 1). Control plants emitted 70 of these VOCs, BT-infested plants 75 VOCs and TA-infested plants 80 VOCs.

**Table 1:** Volatile compounds detected in the headspace of tomato plants without herbivore infestation (C), tomato plants infested with *Bemisia tabaci* (BT) and tomato plants infested with *Tuta absoluta* (TA) according to their elution order in a chromatographic window

No	Compound	Class	Quantifier ion (*m/z*)^A^	LRI_exp._	LRI_lit._	^#^Relative amounts of volatiles (Mean ± SE)^B^		
						C (*N* = *10*)	BT (*N* = *10*)	TA (*N* = *10*)
1	1-Penten-3-ol	Alcohol	57	659	672	^9^81.94 ± 23.78^c^	550.46 ± 163.39^b^	8111.89 ± 2737.95^a^
2	3-Pentanol	Alcohol	59	673	690	308.54 ± 130^c^	947.71 ± 298.06^b^	9857.23 ± 2822.90^a^
3	3-Methylbutan-1-ol	Alcohol	70	713	726	0 ± 0^c^	^6^51.13 ± 30.34^b^	412.87 ± 121.43^a^
4	(*E*)-2-Pentenal	Aldehyde	55	736	745	^4^3.47 ± 1.54^c^	^7^19.35 ± 6.44^b^	^8^516.98 ± 184.34^a^
5	(*Z*)-2-Penten-1-ol	Alcohol	68	760	767	0 ± 0^c^	^6^12.43 ± 4.64^b^	505.93 ± 205.89^a^
6	(*E*)-2-Hexenal	Aldehyde	98	850	850	^4^2.14 ± 0.94^c^	^6^13.51 ± 4.52^b^	1567.24 ± 634.63^a^
7	(*Z*)-3-Hexen-1-ol	Alcohol	82	860	860	^9^152.12 ± 46.94^c^	1363.18 ± 461.23^b^	18494.28 ± 6161.94^a^
8	(*E*,*E*)-2,4-Hexadienal	Aldehyde	81	912	912	^3^4.30 ± 2.54^c^	^6^22.64 ± 8.29^b^	576.28 ± 189.73^a^
9	(*Z*)-2-Penten-1-yl acetate	Ester	68	915	909	0 ± 0^b^	^1^1.19 ± 1.19^b^	^9^312.47 ± 133.17^a^
10	(*Z*)-3-Hexen-1-yl formate	Ester	82	922	920	0 ± 0^b^	^4^6.23 ± 5.96^b^	^7^36.77 ± 14.54^a^
11	(*E*)-4-Oxo-2-hexenal	Aldehyde	55	961	976^C^	^4^13.39 ± 6.09^c^	^8^179.16 ± 63.59^b^	11276.54 ± 4314.56^a^
12	Myrcene	Monoterpene	69	991	991	806.80 ± 665.78^a^	300.90 ± 149.57^a^	^9^821.53 ± 654.77^a^
13	(*Z*)-3-Hexen-1-yl acetate	Ester	82	1008	1008	^4^18.28 ± 8.49^b^	^7^35.01 ± 16.70^b^	^9^5055.68 ± 2544.35^a^
14	α-Phellandrene	Monoterpene	93	1010	1010	^8^2857.38 ± 2579.48^a^	814.26 ± 745.77^a^	^8^3962.29 ± 2580.66^a^
15	α-Terpinene	Monoterpene	93	1021	1021	^9^10891.34 ± 9711.93^a^	^8^2780.76 ± 2499.93^a^	^8^39937.18 ± 32945.11^a^
16	Limonene	Monoterpene	136	1030	1028	^7^17414.13 ± 15480.37^a^	^7^4711.07 ± 4184.55^a^	^7^34953.70 ± 25328.57^a^
17	1,8-Cineole	Monoterpene	154	1032	1032	^9^23.78 ± 11.55^a^	^9^21.27 ± 6.24^a^	^5^22.05 ± 10.68^a^
18	Benzyl alcohol	ar-Alcohol	108	1039	1039	^9^52.69 ± 22.10^b^	^7^44.46 ± 11.95^b^	^8^1370.22 ± 616.30^a^
19	Phenylacetaldehyde	ar-Aldehyde	122	1045	1045	^9^11.74 ± 2.21^b^	^9^18.64 ± 3.55^a,b^	^9^37.88 ± 5.97^a^
20	(*E*)-β-Ocimene	Monoterpene	93	1049	1049	^9^177.06 ± 121.07^b^	295.16 ± 204.10^b^	^9^8875.36 ± 3070.76^a^
21	Conophthorin	Acetal	87	1058	1056^C^	34.29 ± 5.45^b^	71.89 ± 15.86^a,b^	^9^255.92 ± 61.20^a^
22	Terpinolene	Monoterpene	136	1090	1090	229.48 ± 208.94^a^	42.58 ± 27.49^a^	3329.66 ± 3202.90^a^
23	(*Z*)-2-Penten-1-yl butyrate	Ester	68	1091	1089^C^	0 ± 0^b^	^1^1.14 ± 1.14^b^	^9^518.54 ± 294.62^a^
24	Methyl benzoate	ar-Ester	136	1097	1097	^5^7.09 ± 4.41^b^	^7^10.99 ± 6.33^b^	^9^469.09 ± 136.74^a^
25	(*Z*)-3-Hexen-1-yl propanoate	Ester	82	1100	1100	^3^7.99 ± 4.50^c^	^4^11.49 ± 6.05^b,c^	^8^2054.65 ± 1020.32^a^
26	Linalool	Monoterpene	93	1102	1102	^6^18.39 ± 8.22^b^	^6^14.14 ± 7.63^b^	^9^937.43 ± 329.92^a^
27	(*E*)–DMNT	Terpenoid	69	1117	1120^C^	27.33 ± 10.77^b^	^8^43.03 ± 20.57^b^	1286.67 ± 695.97^a^
28	Allo-ocimene	Monoterpene	121	1131	1131	29.14 ± 23.93^b^	^9^17.56 ± 11.26^b,c^	1145.33 ± 1065.68^a^
29	(*E*,*E*)-Cosmene	Monoterpene	134	1132	1134	^1^49.84 ± 49.70^b^	^3^2.60 ± 1.96^b^	104.75 ± 35.88^a^
30	(*Z*)-3-Hexen-1-yl isobutyrate	Ester	82	1145	1144^C^	^4^4.90 ± 2.60^b^	^3^6.67 ± 4.30^b^	1564.66 ± 815.32^a^
31	(*Z*)-3-Hexen-1-yl crotonate	Ester	67	1172	NF	0 ± 0^b^	0 ± 0^b^	^9^875.46 ± 307.81^a^
32	(*Z*)-3-Hexen-1-yl butyrate	Ester	82	1186	1186	^8^128.06 ± 61.32^b^	106.82 ± 33.89^b^	16872.08 ± 6969.29^a^
33	Hexyl butanoate	Ester	89	1192	1192	^7^13.01 ± 5.38^b^	^9^10.27 ± 2.39^b^	853.94 ± 384.73^a^
34	Methyl salicylate	ar-Ester	152	1198	1198	83.76 ± 42.78^c^	775.95 ± 518.59^b^	7545.89 ± 2651.47^a^
35	β-Cyclocitral	Monoterpene	152	1224	1224	^3^0.82 ± 0.53^b^	^9^4.47 ± 1.13^b^	95.65 ± 21.43^a^
36	(*Z*)-3-Hexen-1-yl isovalerate	Ester	82	1233	1230	^4^9.13 ± 4.98^b^	^7^13.17 ± 7.41^b^	1983.95 ± 718.40^a^
37	(*Z*)-3-Hexen-1-yl 2-methylbutanoate	Ester	82	1237	1231^C^	^5^4.07 ± 2.30^b^	^4^3.56 ± 1.59^b^	564.45 ± 185.10^a^
38	Linaloyl acetate	Ester	93	1257	1257	^7^25.48 ± 6.82^a,b^	^7^14.15 ± 7.39^b^	^9^106.43 ± 68.77^a^
39	Piperitone	Monoterpene	110	1258	1258	^6^53.41 ± 45.80^a^	^6^6.32 ± 3.22^b^	^7^28.45 ± 13.29^a^
40	Unknown	NA	83	NA	NA	82.22 ± 17.24^a,b^	56.63 ± 13.34^b^	164.39 ± 33.12^a^
41	(*Z*)-3-Hexen-1-yl valerate	Ester	82	1285	1287^C^	^1^0.45 ± 0.45^b^	0 ± 0^b^	^8^129.94 ± 58.11^a^
42	(*Z*)-3-Hexen-1-yl angelate	Ester	82	1288	NF	0 ± 0^b^	0 ± 0^b^	112.99 ± 56.14^a^
43	Indole	Heterocyclic	117	1299	1300	^8^65.49 ± 25.05^c^	^9^428.94 ± 285.97^b^	11180.01 ± 3527.63^a^
44	(*Z*)-3-Hexen-1-yl tiglate	Ester	67	1326	1322^C^	^8^23.62 ± 10.28^b^	^9^20.83 ± 9.94^b^	1672.61 ± 446.16^a^
45	Methyl anthranilate	ar-Ester	151	1346	1337^C^	^2^1.19 ± 0.91^b^	^1^1.71 ± 1.71^b^	^9^109.48 ± 43.42^a^
46	Benzyl butanoate	ar-Ester	108	1347	1347	^4^2.95 ± 1.89^b^	^3^1.26 ± 0.76^b^	155.15 ± 75.18^a^
47	Eugenol	Phenol	164	1361	1361	^1^0.97 ± 0.97^b^	^1^0.39 ± 0.38^b^	139.85 ± 55.87^a^
48	2-Acetoxypulegone	Ketone	81	1373	NF	^8^59.53 ± 21.43^a,b^	^9^38.71 ± 8.78^b^	^9^128.31 ± 38.35^a^
49	α-Copaene	Sesquiterpene	161	1381	1382	95.67 ± 31.60^c^	1681.38 ± 617.71^a^	125.28 ± 78.25^b^
50	(*Z*)-3-Hexen-1-yl hexanoate	Ester	82	1382	1382	^5^9.55 ± 5.11^b^	^9^33.01 ± 11.50^b^	^9^269.32 ± 119.31^a^
51	(*Z*)-3-Hexen-1-yl (*Z*)-3-hexenoate	Ester	82	1386	1383^C^	^2^2.70 ± 1.88^b^	0 ± 0^b^	^8^120.81 ± 50.87^a^
52	β-Elemene	Sesquiterpene	93	1396	1397	^5^16.97 ± 15.06^b^	58.46 ± 24.34^a,b^	^6^71.65 ± 59.36^a^
53	(*Z*)-Jasmone	Ketone	164	1402	1403	^9^64.28 ± 36.81^b^	14.15 ± 6.96^b^	421.42 ± 124.56^a^
54	Unknown	ar-Unknown	150	NA	NA	0 ± 0^b^	0 ± 0^b^	^8^28.33 ± 11.48^a^
55	(*E*)-β-Caryophyllene	Sesquiterpene	93	1428	1428	592.77 ± 568.16^a,b^	^9^249.65 ± 223.20^b^	^8^2569.85 ± 2401.01^a^
56	(*E*)-α-Ionone	Terpenoid	121	1432	1432	^4^2.15 ± 0.92^b^	^2^1.09 ± 0.74^b^	^8^13.15 ± 5.25^a^
57	β-Copaene	Sesquiterpene	161	1435	1435	9.26 ± 3.16^b^	113.23 ± 43.18^a^	^9^12.42 ± 5.13^b^
58	α-Caryophyllene	Sesquiterpene	93	1461	1461	^4^301.89 ± 290.46^a^	^5^116.60 ± 105.07^a^	^5^1522.66 ± 1429.05^a^
59	Valencene	Sesquiterpene	161	1484	1484	17.84 ± 8.73^b^	72.64 ± 22.97^a^	30.33 ± 8.93^b^
60	Bicyclosesquiphellandrene	Sesquiterpene	161	1488	1471	^5^15.85 ± 13.58^b^	^8^19.43 ± 12.05^b^	^3^81.46 ± 75.06^a^
61	(*E*)-β-Ionone	Terpenoid	177	1490	1490	28.28 ± 10.24^b^	37.63 ± 8.00^b^	576.25 ± 93.82^a^
62	Aristolochene	Sesquiterpene	189	1494	1487^C^	^4^2.97 ± 2.03^b^	332.87 ± 222.65^a^	^7^2.56 ± 0.77^b^
63	β-Chamigrene	Sesquiterpene	189	1502	1503	^3^5.80 ± 5.43^a^	^7^8.87 ± 2.81^a^	^3^5.75 ± 3.91^a^
64	Patchoulene	Sesquiterpene	161	1506	1484	^7^4.23 ± 1.93^b^	17.67 ± 6.08^a^	^7^7.41 ± 2.94^ab^
65	(*E*,*E*)-α-Farnesene	Sesquiterpene	93	1509	1509	^2^2.72 ± 2.12^b^	^7^11.78 ± 4.65^b^	^9^84.06 ± 23.47^a^
66	Unknown	NA	107	NA	NA	^5^4.09 ± 2.00^b^	^8^26.89 ± 13.05^a,b^	^9^53.19 ± 16.11^a^
67	(*Z*)-3-Hexen-1-yl benzoate	Ester	82	1574	1575	^7^94.48 ± 48.53^b^	^7^48.59 ± 11.41^b^	942.74 ± 360.51^a^
68	(*E*,*E*)-TMTT	Terpenoid	81	1582	1589^C^	965.38 ± 267.09^b^	4286.68 ± 1887.88^a,b^	9157.15 ± 2776.07^a^
69	Methyl *cis*-dihydrojasmonate	Ester	156	1657	1654^C^	90.42 ± 26.17^a^	82.31 ± 13.80^a^	149.24 ± 35.69^a^
70	Unknown	NA	119	NA	NA	581.84 ± 206.18^a^	402.81 ± 162.87^a^	981.60 ± 231.46^a^
71	IPDMOHM	Sesquiterpene	191	1679	1659	348.57 ± 120.43^a^	242.89 ± 93.41^a^	607.50 ± 126.71^a^
72	Unknown	NA	191	NA	NA	52.02 ± 17.21^a,b^	37.58 ± 13.85^b^	91.67 ± 16.47^a^
73	Unknown	NA	135	NA	NA	152.73 ± 51.65^a^	104.92 ± 42.65^a^	242.80 ± 54.18^a^
74	Unknown	NA	232	NA	NA	4.05 ± 0.88^a^	^8^9.41 ± 2.78^a^	12.89 ± 6.55^a^
75	Unknown	NA	232	NA	NA	^9^4.01 ± 0.88^a^	8.09 ± 2.05^a^	12.38 ± 6.54^a^
76	Unknown	NA	232	NA	NA	^9^2.96 ± 0.59^a^	6.93 ± 1.89^a^	11.54 ± 6.56^a^
77	4-Acetyl-α-cedrene	Ketone	161	1779	NF	297.11 ± 105.52^a^	268.11 ± 66.43^a^	417.32 ± 105.83^a^
78	Unknown	NA	246	NA	NA	0 ± 0^b^	^4^3.83 ± 1.84^a^	^3^5.53 ± 4.27^a^
79	Unknown	NA	246	NA	NA	0 ± 0^b^	^4^4.29 ± 1.99^a^	^1^3.88 ± 3.87^a^
80	Unknown	NA	246	NA	NA	^3^2.12 ± 1.23^a^	^5^4.92 ± 1.92^a^	^5^6.55 ± 3.87^a^

Significant differences in the volatile emissions among plants exposed to three treatments based on the Kruskal Wallis non-parametric test exist when means have no superscript letters in common

LRI_Exp._: Linear retention indices experimentally obtained on a ZB-5MSi analytical column

LRI_Lit._: Linear retention indices obtained from NIST 2008, on a column with (5%-Phenyl)-methylpolysiloxane stationary phase or equivalent unless stated otherwise

NA: Not Applicable

NF: LRI_Lit._ Not Found

ar: aromatic volatile

(*E*)–DMNT: (*E*)-4,8-dimethylnona-1,3,7-triene

(*E*, *E*)–TMTT: (*E*, *E*)-4,8,12-trimethyltrideca-1,3,7,11-tetraene

IPDMOHM: (7a–Isopropenyl-4,5-dimethyloctahydroinden-4-yl)methanol

^A^Quantifier ion used for relative quantification of the respective volatile compounds

^B^Relative amounts of volatile compound emitted from control plants (C), plants infested with *B. tabaci* (BT) or *T. absoluta* (TA) using a single quantifier (target) ion are given as mean peak area ± SE per gram fresh weight of foliage divided by 10^3^. The number of replicates for each treatment is given in parentheses

^C^LRI_Lit._ obtained from Adams (1995), Citron et al. (2012), Kos et al. (2013), Marques et al. (2007), Ruther (2000), and Zeng et al. (2016)

^**#**^Numbers in superscript before the emission quantities represent the number of samples in which a given compound was detected and quantified

Qualitative differences were found for three VOCs (**31, 42, 54**) that only occurred in headspace samples from TA-infested plants. There was variability in the presence of some compounds even within the same treatment groups, where some compounds were only detected in one or two samples of the same treatment, especially in the control and BT-infested plant samples. Therefore, we used consistency of occurrence, here defined as occurrence in minimally 70% of the samples, as an additional criterion for qualitative differences between treatments, resulting in 10 compounds (**3, 5, 9, 10, 23, 31, 41, 42, 51,** & **54**), most of which are volatile metabolites of C_18_-fatty acids that were consistently found only in the samples of the TA-infested plants compared to control and BT-infested plants.

Major quantitative differences were found for many VOCs among plants exposed to one of the two herbivory treatments (Table 1). More than half of the listed volatiles were emitted at significantly higher levels by plants exposed to the tomato borer *T. absoluta* when compared to either intact undamaged plants or those treated with *B. tabaci* whiteflies (*Kruskal Wallis test*; *P* < 0.001). These compounds typically comprise volatile metabolites of C_18_-fatty acids (C_5_- and C_6_-compounds including “green leaf volatiles” and jasmone), aromatic volatiles derived from chorismate such as benzyl alcohol, methyl salicylate, methyl anthranilate, benzyl butanoate, and eugenol; terpenoids – acyclic: [(*E*)-β-ocimene, linalool, allo-ocimene, (*E,E*)-cosmene, (*E,E*)-α-farnesene, and (*E*)-DMNT] and cyclic [(*E*)-α- and β-ionone]. In contrast, some cyclic sesquiterpenes such as α- and β-copaene, valencene, and aristolochene were released at significantly higher levels from the plants infested with the phloem-sucking whitefly *B. tabaci*. No significant differences in levels of cyclic monoterpenes were found between the treatments except for β-cyclocitral, the emission level of which was significantly higher in TA-infested plants.

**Fig. 1:**
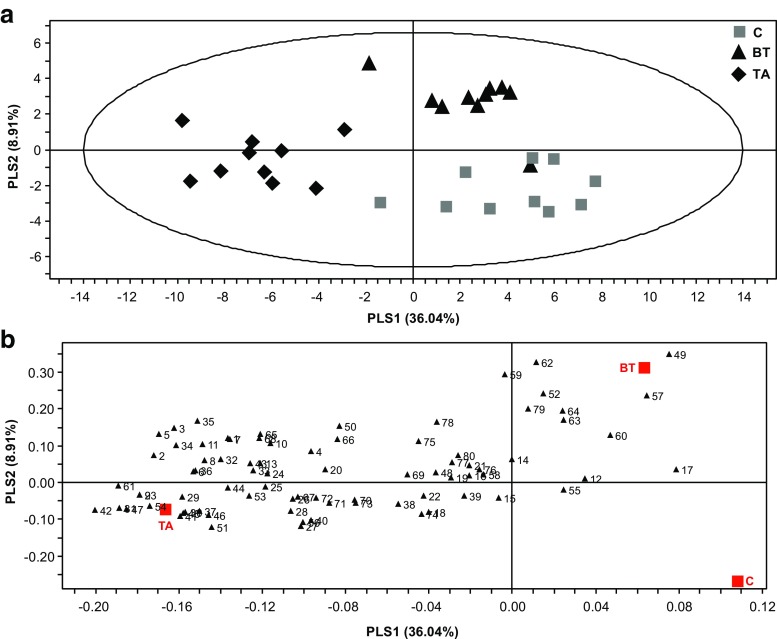
Graphical representation of projection to latent structures-discriminant analysis (PLS-DA) applied on headspace composition of tomato plants infested with *Tuta absoluta* (TA, *N* = 10) or *Bemisia tabaci* (BT, *N* = 10) or with no infestation as the control (C, *N* = 10). Score plot (**a**) visualizing the grouping pattern of the samples according to the first two principal components (PCs) with the explained variance in parenthesis. The contribution of each volatile compound to the group separation is displayed in the loading plot (**b**). For compound identity in relation to the numbering in the loading plot, please refer to Table 1

Projection to latent structures discriminant analysis (PLS-DA) of all treatments together presented three major clusters of samples, where the two herbivory treatments were separated from the undamaged control plants and from each other (Fig. 1a). The separation was influenced mainly by the herbivore treatment, where the C_5_ and C_6_-compounds, chorismate-derived aromatic compounds, and terpenoids (mostly acyclic ones) were highly correlated with *T. absoluta* infestation, whereas cyclic sesquiterpenes were highly correlated with *B. tabaci-*infested plants. Among the 80 headspace volatiles used for this analysis, 38 contributed most to the separation between the treatments, with variable importance for the projection (VIP) values >1 (Table 2). These compounds included volatile metabolites of C_18_-fatty acids and branched chain amino acids: **3, 42, 5, 31, 2, 51, 9, 23, 41, 11, 1, 7, 30, 50, 37, 8, 6, 36,** & **10**; aromatic volatiles: **47, 34, 45,** & **46**; terpenoids: **49, 62, 59, 57, 52, 35, 61, 64, 65, 68, 63, & 29**; and unknowns: **79, 54,** & **78**. The correlation between the contributions of these compounds with at least one of the three treatments is clearly visible from the loading plot (Fig. 1b).

**Table 2:** Values of Variable Importance to the Projection (VIP) of volatile compounds for the corresponding PLS-DA plots (Figs. 1, 2, 3) based on the headspace composition of tomato plants subjected to: *Tuta absoluta* infestation (TA, *N* = 10) or *Bemisia tabaci* infestation (BT, *N* = 10) or no infestation as the control (C, *N* = 10) of tomato plants. Compounds are listed according their elution order in a chromatographic window

^a^No	Compound	^b^PLS-DA (C, TA & TB)	^c^PLS-DA (C vs BT)	^d^PLS-DA (C vs TA)
1	1-Penten-3-ol	**1.16**	**1.20**	**1.15**
2	3-Pentanol	**1.21**	**1.43**	**1.41**
3	3-Methylbutan-1-ol	**1.40**	**1.63**	**1.65**
4	(*E*)-2-Pentenal	0.81	**1.06**	**1.00**
5	(*Z*)-2-Penten-1-ol	**1.37**	**1.63**	**1.64**
6	(*E*)-2-Hexenal	**1.01**	0.95	**1.34**
7	(*Z*)-3-Hexen-1-ol	**1.15**	**1.16**	**1.16**
8	(*E*,*E*)-2,4-Hexadienal	**1.03**	0.91	**1.39**
9	(*Z*)-2-Penten-1-yl acetate	**1.17**	-	**1.43**
10	(*Z*)-3-Hexen-1-yl formate	**1.00**	**1.25**	**1.16**
11	(*E*)-4-Oxo-2-hexenal	**1.16**	**1.17**	**1.35**
12	β-Myrcene	0.23	0.38	0.24
13	(*Z*)-3-Hexen-1-yl acetate	0.85	0.96	**1.07**
14	α-Phellandrene	0.39	0.63	0.10
15	α-Terpinene	0.26	0.60	0.05
16	Limonene	0.18	0.37	0.30
17	1,8-Cineole	0.55	0.34	0.58
18	Benzyl alcohol	0.55	0.52	0.51
19	Phenylacetaldehyde	0.21	0.28	0.28
20	(*E*)-β-Ocimene	0.62	0.70	0.71
21	Conophthorin	0.32	**1.32**	0.20
22	Terpinolene	0.35	0.41	0.33
23	(*Z*)-2-Penten-1-yl butyrate	**1.17**	-	**1.46**
24	Methyl benzoate	0.77	0.62	**1.03**
25	(*Z*)-3-Hexen-1-yl propanoate	0.77	0.84	**1.00**
26	Linalool	0.72	0.23	0.87
27	(*E*)–DMNT	0.98	0.71	**1.19**
28	Allo-ocimene	0.83	0.33	0.99
29	(*E*,*E*)-Cosmene	**1.05**	0.56	**1.36**
30	(*Z*)-3-Hexen-1-yl isobutyrate	**1.12**	0.84	**1.29**
31	(*Z*)-3-Hexen-1-yl crotonate	**1.29**	-	**1.45**
32	(*Z*)-3-Hexen-1-yl butyrate	0.99	0.85	**1.12**
33	Hexyl butanoate	0.83	0.56	**1.06**
34	Methyl salicylate	**1.22**	**1.54**	**1.36**
35	β-Cyclocitral	**1.43**	**1.62**	**1.37**
36	(*Z*)-3-Hexen-1-yl isovalerate	**1.01**	0.84	**1.30**
37	(*Z*)-3-Hexen-1-yl 2-methylbutanoate	**1.07**	0.91	**1.27**
38	Linaloyl acetate	0.49	0.37	0.40
39	Pipertone	0.26	0.32	0.53
40	Unknown	0.87	0.56	0.72
41	(*Z*)-3-Hexen-1-yl valerate	**1.16**	-	**1.15**
42	(*Z*)-3-Hexen-1-yl angelate	**1.37**	-	**1.59**
43	Indole	0.88	0.66	**1.12**
44	(*Z*)-3-Hexen-1-yl tiglate	0.88	0.68	**1.13**
45	Methyl anthranilate	**1.14**	0.84	**1.22**
46	Benzyl butanoate	**1.08**	0.89	**1.24**
47	Eugenol	**1.27**	-	**1.48**
48	2-Acetoxypulegone	0.29	0.30	0.53
49	α-Copaene	**2.20**	**2.21**	0.42
50	(*Z*)-3-Hexen-1-yl hexanoate	**1.09**	**1.23**	0.98
51	(*Z*)-3-Hexen-1-yl (*Z*)-3-hexenoate	**1.19**	-	**1.11**
52	β-Elemene	**1.50**	**1.67**	0.39
53	(*Z*)-Jasmone	0.84	0.58	**1.03**
54	Unknown	**1.19**	-	**1.31**
55	(*E*)-β-Caryophyllene	0.20	0.34	0.33
56	(*E*)-α-Ionone	0.92	0.66	0.74
57	β-Copaene	**1.51**	**2.11**	0.21
58	α-Caryophyllene	0.16	0.32	0.35
59	Valencene	**1.81**	**1.85**	0.61
60	Bicyclosesquiphellandrene	0.85	0.92	0.21
61	(*E*)-β-Ionone	**1.22**	0.85	**1.48**
62	Aristolochene	**2.02**	**2.10**	0.93
63	β-Chamigrene	**1.06**	**1.07**	0.21
64	Patchoulene	**1.20**	**1.47**	0.51
65	(*E*,*E*)-α-Farnesene	**1.14**	**1.27**	**1.22**
66	Unknown	0.91	**1.00**	0.93
67	(*Z*)-3-Hexen-1-yl benzoate	0.70	0.77	0.97
68	(*E*,*E*)-TMTT	**1.09**	**1.33**	**1.21**
69	Methyl *cis*-dihydrojasmonate	0.35	0.46	0.66
70	Unknown	0.56	0.14	0.79
71	IPDMOHM	0.66	0.17	0.81
72	Unknown	0.65	0.04	0.83
73	Unknown	0.59	0.23	0.78
74	Unknown	0.59	0.48	0.73
75	Unknown	0.75	0.74	0.50
76	Unknown	0.25	0.32	0.59
77	4-Acetyl-α-cedrene	0.38	0.47	0.79
78	Unknown	**1.04**	**1.25**	0.80
79	Unknown	**1.24**	**1.25**	-
80	Unknown	0.49	0.64	0.54

Bold face type scores are higher than 1 and are most influential for separation of the treatments in a given PLS-DA model

**Fig. 2:**
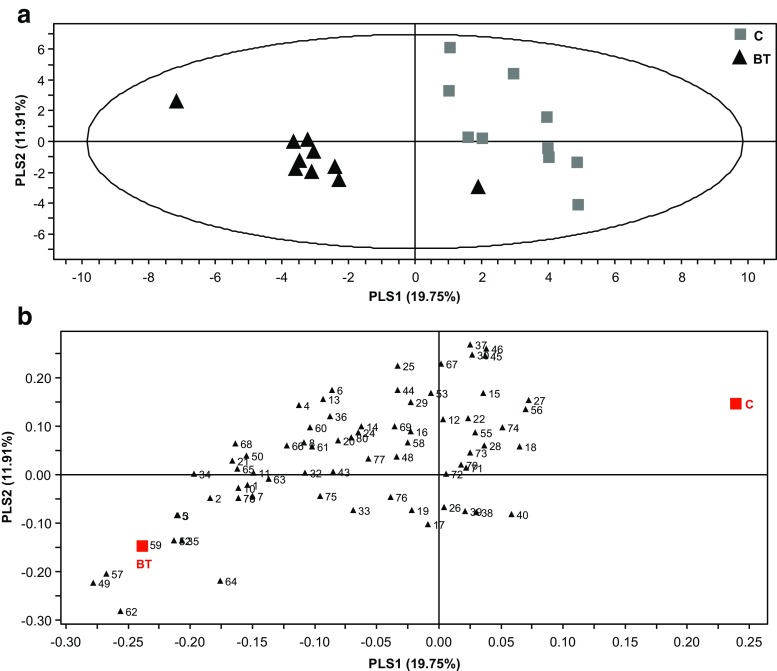
Graphical representation of projection to latent structures-discriminant analysis (PLS-DA) applied on the headspace composition of tomato plants infested with *Bemisia tabaci* (BT, *N* = 10) and non-infested control plants (*C*, *N* = 10) (**a**). The contribution of each volatile to the group separation is displayed in their corresponding loading plots (**b**). For compound identity in relation to the numbering in the loading plots, please refer to Table 1

**Fig. 3:**
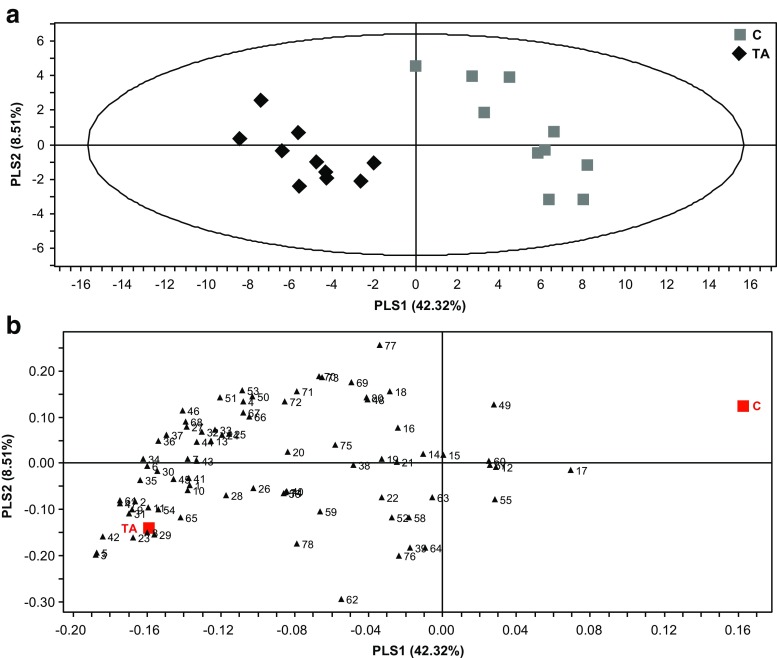
Graphical representation of projection to latent structures-discriminant analysis (PLS-DA) applied on headspace composition of tomato plants infested with *Tuta absoluta* (TA, *N* = 10) and non-infested control plants (*C*, *N* = 10) (**a**). The contribution of each volatile to the group separation is displayed in their corresponding loading plots (**b**). For compound identity in relation to the numbering in the loading plots, please refer to Table 1

^a^Compound numbering corresponds to the loading plots in Figs. 1, 2, and 3

^b^VIP values obtained during PLS-DA analysis of all treatments together (Fig. 1)

^c^VIP values obtained during PLS-DA analysis of BT infested and control plants (Fig. 2a, b)

^d^VIP values obtained during PLS-DA analysis of TA infested and control plants (Fig. 3a, b)

A detailed analysis of the compositional differences between the HIPV-blends emitted by plants infested by either herbivore and the control plants was carried out. PLS-DA analysis yielded a clear separation between BT-infested and control plants (Fig. 2a). In total, 24 compounds contributed most to the separation (Fig. 2b) based on VIP values higher than 1. Listed with numbers in the order of decreasing VIP-value these compounds are: **49, 57, 62, 59, 52, 5, 3, 35, 34, 64, 2, 68, 21, 65, 10, 78, 79, 50, 1, 11, 7, 63**, **4, & 66** (Tables 1, 2; Fig. 2b). All these compounds were positively correlated to the *B. tabaci* infested tomato plants (Fig. 2b), and were emitted in elevated amounts when compared to uninfested plants.

A similar pairwise PLS-DA analysis between *T. absoluta-*infested and uninfested plants showed a clear separation of the treatment groups based on the composition of their headspace volatiles (Fig. 3a). The PLS-DA analysis identified 38 compounds with a VIP value higher than 1. These compounds are dominated by the volatile metabolites of C_18_-fatty acids and branched chain amino acids (in Tables 1, 2; Fig. 3b; compound numbers: **1**–**11**, **13**, **23**, **25**, **30**–**33**, **36**, **37**, **41**, **42**, **44**, **51,** & **53**), chorismate-derivatives (in Tables 1, 2; Fig. 3b; compound numbers: **24**, **34**, **43**, **45**, **46,** & **47**), terpenoids: **27**, **29**, **35**, **61**, **65,** & **68**), and an unknown: **54**. In addition, (*Z*)-2-penten-1-yl acetate (**9**) and (*Z*)-2-penten-1-yl butyrate (**23**), were detected in the headspace of *T. absoluta* treated plants and in only one sample of *B. tabaci* treated plants, while (*Z*)-3-hexen-1-yl (*E*)-2-butenoate (**31**) and (*Z*)-3-hexen-1-yl 2-methyl-2-butenoate (**42**) were detected only in the headspace of *T. absoluta* treated plants (VIP > 1, Table 1).

## Discussion

### Herbivore Feeding Mode and Signal Transduction Pathways in VOC Biosynthesis

Plants synthesize and release an array of VOCs derived from a diverse set of primary metabolites that include amino acids, fatty acids, and sugars (Schwab et al. 2008). These volatiles have a range of functions in intra- and inter-kingdom interactions, including those among plants and insects (Dicke and Baldwin 2010). Immediately upon damage by biting-chewing herbivores such as TA, tomato plants show enhanced emission of volatile metabolites of fatty acids, which are the result of the breakdown of lipids through the lipoxygenase (LOX) pathway (Shen et al. 2014). Breakdown of plant cell membranes gives rise to free linoleic and/or linolenic acid, both of which are acted upon by LOX to form C_5_ volatile compounds and the C_6_ green leaf volatiles (Croft et al. 1993; McCormick et al. 2012; Shen et al. 2014). Similarly, volatiles likely derived from branched chain amino acids such as valine, leucine, and isoleucine (Gonda et al. 2010; Kochevenko et al. 2012) show immediate induction and measured at higher level upon infestation with TA herbivores.

Biting-chewing and piercing-sucking insects elicit distinct defense pathways in plants (Kempema et al. 2007; Walling 2000; Zhang et al. 2009, 2013). HIPV emission is known to be mainly regulated by the octadecanoid or JA signal-transduction pathway (Ament et al. 2004). Piercing-sucking insects such as whiteflies and aphids predominantly activate the SA signaling pathway (Kempema et al. 2007; Stam et al. 2014; Zarate et al. 2007). In the present study, the level of methyl salicylate, a volatile derivative of SA, was higher when tomato plants were infested by TA. Methyl salicylate biosynthesis can be induced downstream of the JA-cascade after attack by chewing herbivores (Ament et al. 2004; Cardoza et al. 2002; Dicke et al. 1999; Rodriguez-Saona et al. 2001). Our results highlight the differential induction of plant volatiles depending on insect feeding mode, where the biting-chewing *T. absoluta* induced both a higher number and higher amounts of HIPVs released from tomato plants than the phloem sucking whitefly *B. tabaci*.

### Qualitative Differences Between Tomato VOC Blends

Volatiles that have been detected only in plants infested by herbivores may be regarded as universal signs of herbivore damage (Schoonhoven et al. 2005). In addition, we found qualitative differences among the herbivory treatments. Ten compounds were consistently detected only in the headspace of TA-infested plants compared to that of control plants, most of which were volatile metabolites of fatty acids and aromatic compounds. Presence / absence differences between VOC blends could have been important for mirid females in discriminating between odor blends emitted by infested and uninfested tomato plants (Lins et al. 2014; De Backer et al. 2015). These volatile metabolites of C_18_-fatty acids were not found in the headspace of tomato plants infested with the whitefly *Trialeurodes vaporariorum* (Westwood) (López et al. 2012), probably due to the fact that phloem feeding insects do not cause damage to plant tissues (Walling 2008). Mono- and sesquiterpenes of the headspace composition of BT-infested plants are qualitatively similar to those detected in the headspace of tomato plants infested with the whitefly *T. vaporariorum* and the aphid *Myzus persicae* (Sulzer) (Errard et al. 2015; López et al. 2012). In another report by Fang et al. (2013), five terpenes from the headspace of BT-infested plants were in agreement with the headspace of BT-infested plants described in this study.

Chemical analysis of the headspace of uninfested and infested tomato plants in this study provided largely different results to previous studies (Degenhardt et al. 2010; Proffit et al. 2011) with very small similarities on the nature of VOCs observed. Furthermore, we did not find two monoterpenes (carene and α-pinene), which were consistently reported in the headspace of tomato plants (Degenhardt et al. 2010; Fang et al. 2013; López et al. 2012; Megido et al. 2014; Proffit et al. 2011; Strapasson et al. 2014). Here, we document the detection of 46 VOCs that have not been found in previous studies on tomato : **1–5, 9–11, 13, 17, 19, 21, 23–25, 29–31, 35–39, 41–46, 48, 50, 51, 53, 56, 57, 59–65, 67, 69, 71, & 77** (Table 1). In addition, De Backer et al. (2015) reported six monoterpene compounds in the headspace of TA-infested tomato plants that were not found in our study. Differences between studies in the emitted blend may be explained by plant cultivar, growing conditions, duration of herbivore infestation as well as by herbivore stage/s and density of infesting the plant, prior to volatile collection (Dudareva et al. 2006; Niinemets et al. 2013).

### Quantitative Differences Between VOC-Blends

TA-infested plants released several compounds in higher amounts than BT-infested plants. These compounds include volatile metabolites of fatty acids and branched chain amino acids such as the C_5_ compounds and the C_6_ green leaf volatiles, JA derivatives: (*Z*)-jasmone and methyl *cis*-dihydrojasmonate (Table 1), as well as terpenoids (**20, 26–29, 65, & 68**). These HIPVs also have been reported to be emitted in increased amounts when other plants are damaged by other biting-chewing insects (Poelman et al. 2012; Ponzio et al. 2013; Vuorinen et al. 2004; War et al. 2011; Weldegergis et al. 2015; Zhang et al. 2013) or when mechanically wounded leaves have been treated with oral secretions of herbivores (Zebelo et al. 2014). These compounds play a role in the attraction of natural enemies such as parasitoids, predatory mites and lacewings (Bukovinszky et al. 2005; Dicke et al. 1990; Smid et al. 2002; War et al. 2011). Strikingly, cyclic sesquiterpenes were the only class of volatiles that were strongly associated with BT-infested plants, and contributed importantly to separating them from the TA-infested and intact control samples. Gosset et al. (2009) reported higher levels of cyclic sesquiterpenes from potato plants (*Solanum tuberosum* L.) when infested by the aphid *Myzus persicae* Sulzer, a piercing-sucking insect, compared to plants infested by the leaf-chewing Colorado potato beetle *Leptinotarsa decemlineata* Say.

Another class of importance in revealing the difference between treatments worth looking at is that of the aromatic volatiles, the role of which in insect-plant interactions is often overlooked. In our study, their release was strongly induced by *T. absoluta* feeding damage. These compounds (methyl benzoate, methyl salicylate, indole, methyl anthranilate, benzyl butanoate, and eugenol) are formed from chorismate or phenylalanine via multiple biosynthetic steps (Dudareva et al. 2006). They were found to occur at significantly higher levels in the emissions of TA-infested plants. The latter three were occurring in the headspace of TA-infested plants at levels 50–250 times higher than in samples from control or BT-infested plants. The emission of most of these volatiles is often associated with flowers and to a lesser extent with leaves (Dudareva et al. 2004), and they are known as defensive chemicals.

### HIPV Blend Composition and Behavioral Discrimination by Mirid Predators

Insects respond according to the blend of volatiles perceived (Bruce and Pickett 2011; De Boer et al. 2004; Dicke et al. 2009; Lins et al. 2014; Moayeri et al. 2007b). Besides the time and energy costs of searching, and the increased likelihood of being preyed while searching, predatory arthropods have to deal with variability in HIPV, emitted by the food plants of their prey.

A previous behavioral study demonstrated that *N. tenuis* and *M. pygmaeus* were attracted to volatile blends released by tomato plants infested by *T. absoluta* and *B. tabaci* (Lins et al. 2014). As a follow-up, we here present volatile emissions of tomato plants after exposure to these two herbivorous pests in order to evaluate the role of HIPVs in enhancing the efficiency of the mirids as biological control agents.

The VOC data reported here can be linked to the findings of our previous behavioral studies in the tritrophic system tomato – herbivore - mirid predator. The VOC profiles of tomato plants infested by the two herbivores differed both qualitatively and quantitatively. Investigation of the chemosensory response, e.g., by electroantennography, of the mirid predators to each compound identified in the HIPV blends emitted from tomato may be used for identification of those HIPVs that contribute to attraction of mirid predators. The lack or presence of particular compounds in the VOC blend can make the plant unrecognizable for naive predators, and learning can be necessary to enhance responses and motivate predators and/or parasitoids to search. Accordingly it was evident that learning by *M. pygmaeus* improved its capacity to find prey (Lins et al. 2014). Insect learning is a well-known and widely studied experience-based modification of behavior (De Boer et al. 2005; Glinwood et al. 2011; Rim et al. 2015; Steidle and Van Loon 2003), however, it was studied only recently for predatory mirid bugs (Lins et al. 2014). Although the C_6_-GLV related compounds were not found in the headspace of BT-infested plants, experienced *N. tenuis* and *M. pygmaeus* were able to discriminate the HIPV-blend of BT-infested plants over those of clean plants (Lins et al. 2014).

In summary, our findings show that feeding by the biting-chewing larvae of the lepidopteran *T. absoluta* and the phloem-sucking *B. tabaci* whiteflies induced quantitatively and qualitatively different HIPV blends. Knowledge about orientation mechanisms of mirid predators is limited and deserves to be studied more extensively as they play an important role in the biological system. Information on the identification of behaviorally active HIPVs and on the phenotypic plasticity in behavioral responses of mirids will contribute to the development of strategies based on semiochemical to improve existing pest control approaches of these tomato pests.

## Electronic supplementary material

Online Resource 1 Supplemental material (SM) 1 A representative total ion chromatogram (TIC) of a typical tomato plant infested with *Tuta absoluta* (TA), portraying the detected volatiles in the given sample. Due to the observed variability among samples including those under the same treatments, compound numbers: 4, 25, 60, 63, 78, 79, and 80 are not detected in the presented sample. For the identity of the numbered peaks please refer to Table 1. It must be noted that the unnumbered extra peaks in the chromatogram correspond to the “background noise” originating from compressed air, glass jars, pots, and/or soils, cleaned Tenax TA adsorbents and the analytical system itself. (PDF 51 kb)

Online Resource 2 Supplemental material (SM) 2 70 eV EI-mass spectra of unknown compounds 40, 54, 66, 70, 72–76, and 78–80 listed in Table 1. (PDF 74 kb)

(PDF 81 kb)

(PDF 65 kb)

(PDF 67 kb)
