# Supplementary figures and images for: Qualitative and Quantitative Differences in Herbivore-Induced Plant Volatile Blends from Tomato Plants Infested by Either Tuta absoluta or Bemisia tabaci
Source: J Chem Ecol. 2017 Jan 3;43(1):53–65. doi: 10.1007/s10886-016-0807-7 (PMC5331093; doi:10.1007/s10886-016-0807-7)

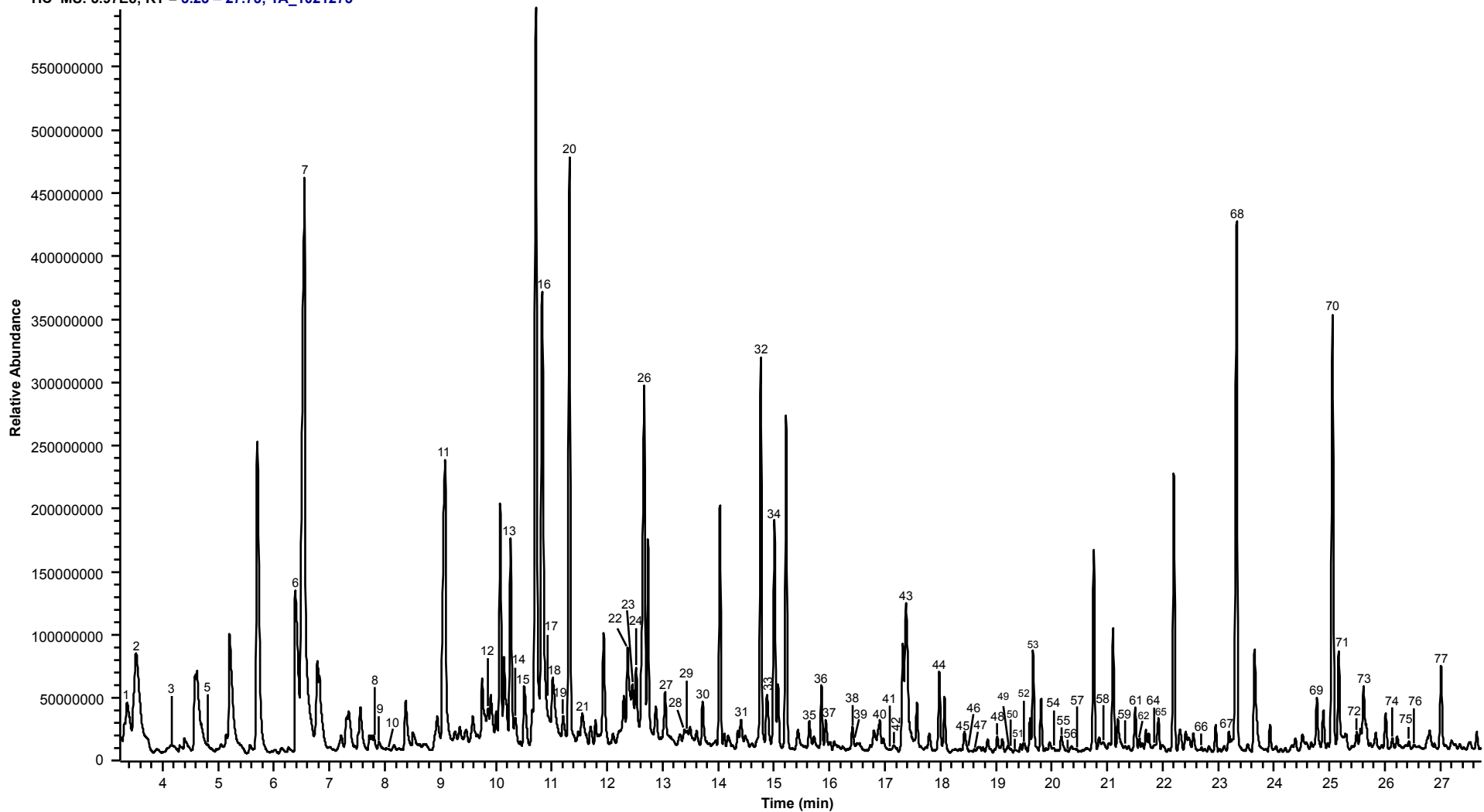

Supplement: Supplementary file 1 — Supplemental material (SM) 1 A representative total ion chromatogram (TIC) of a typical tomato plant infested with Tuta absoluta (TA), portraying the detected volatiles in the given sample. Due to the observed variability among samples including those under the same treatments, compound numbers: 4, 25, 60, 63, 78, 79, and 80 are not detected in the presented sample. For the identity of the numbered peaks please refer to Table 1. It must be noted that the unnumbered extra peaks in the chromatogram correspond to the “background noise” originating from compressed air, glass jars, pots, and/or soils, cleaned Tenax TA adsorbents and the analytical system itself. (PDF 51 kb) [file 10886_2016_807_MOESM1_ESM.pdf]

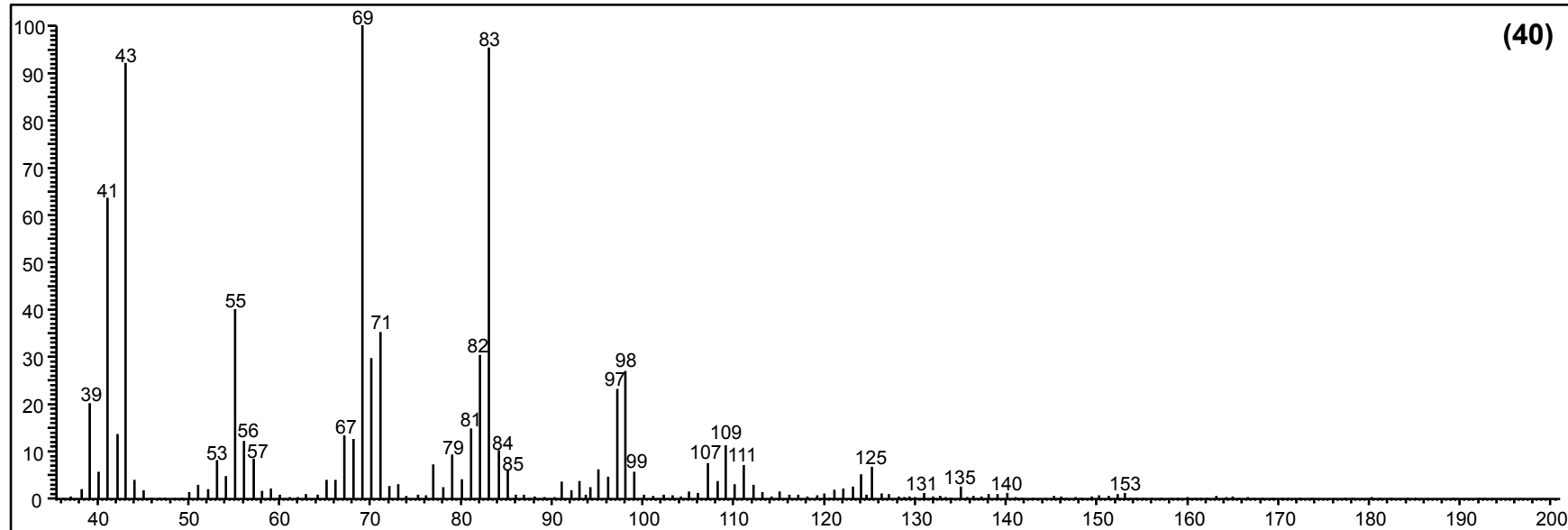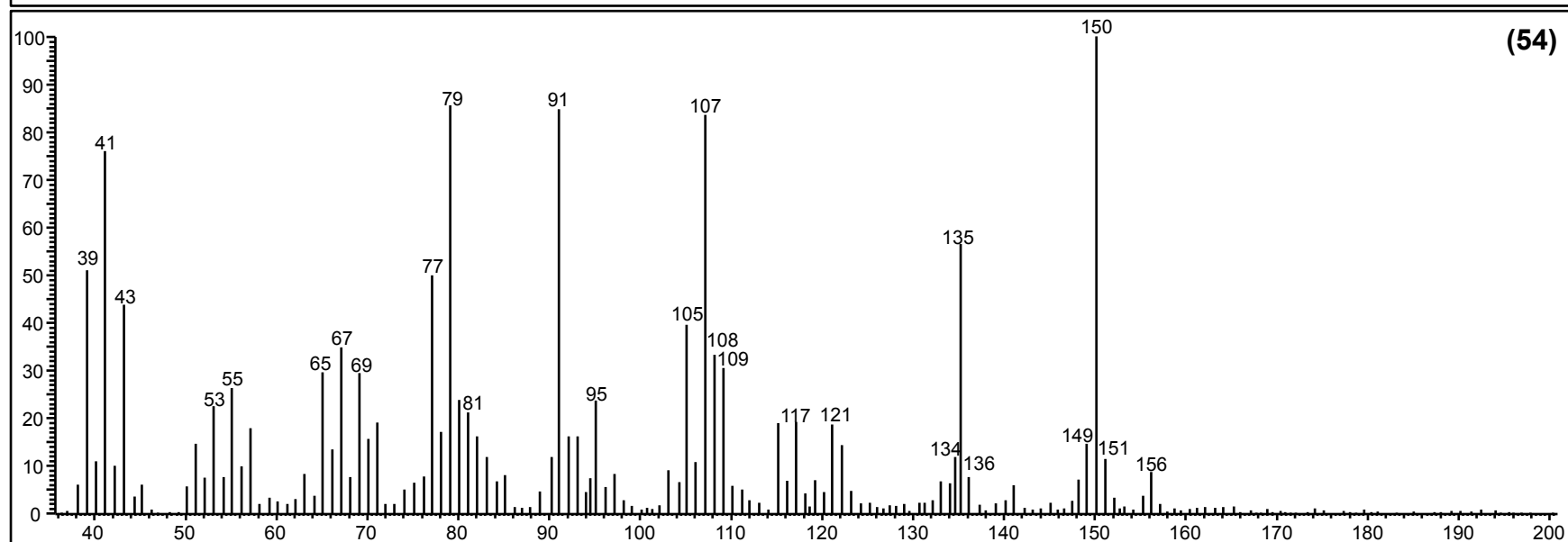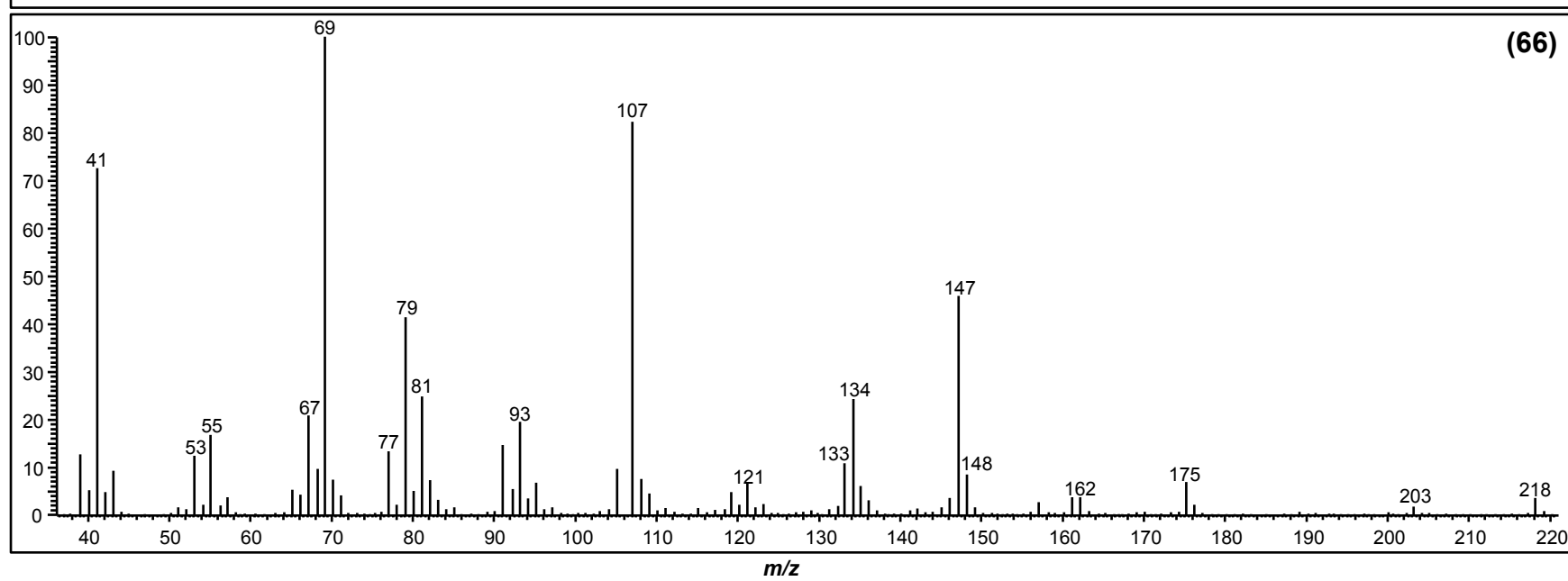

Supplement: Supplementary file 2 — Supplemental material (SM) 2 70 eV EI-mass spectra of unknown compounds 40, 54, 66, 70, 72–76, and 78–80 listed in Table 1. (PDF 74 kb) [file 10886_2016_807_MOESM2_ESM.pdf]

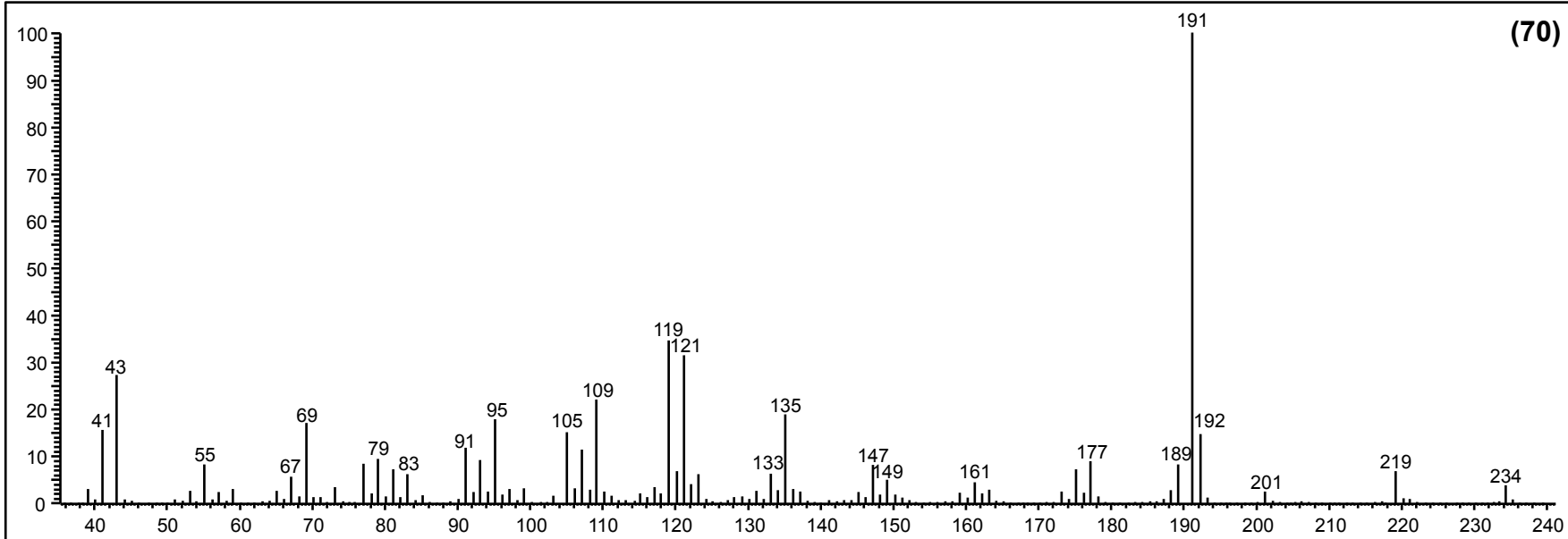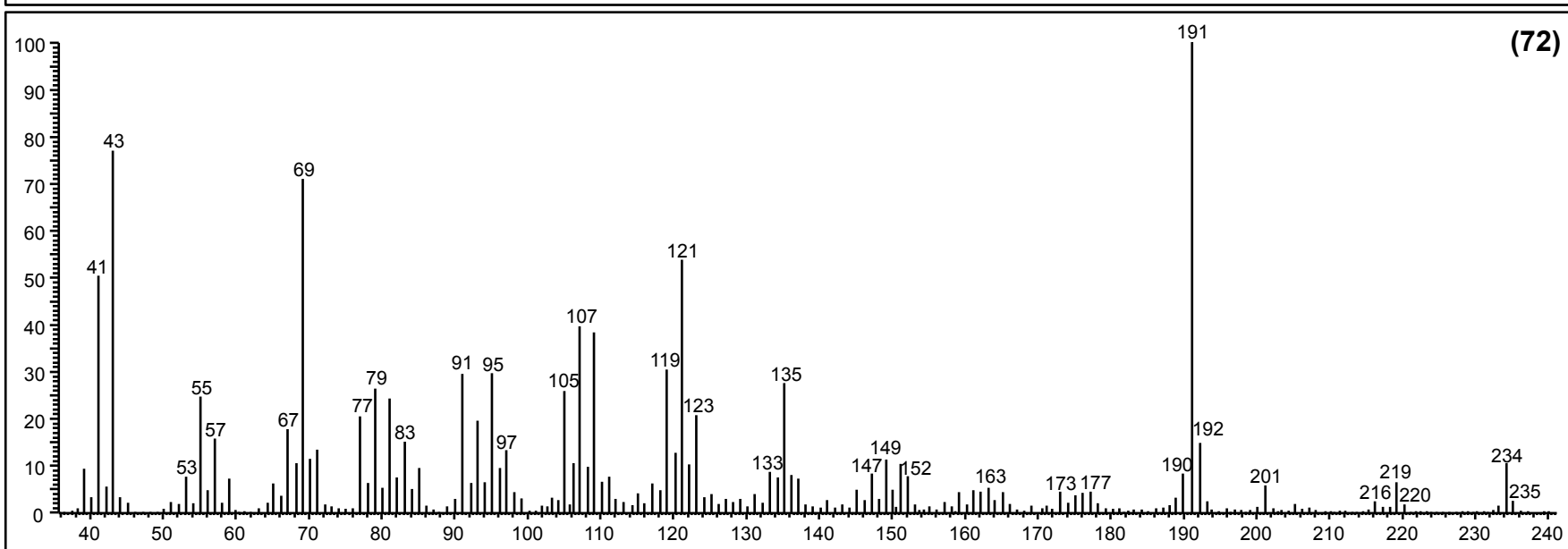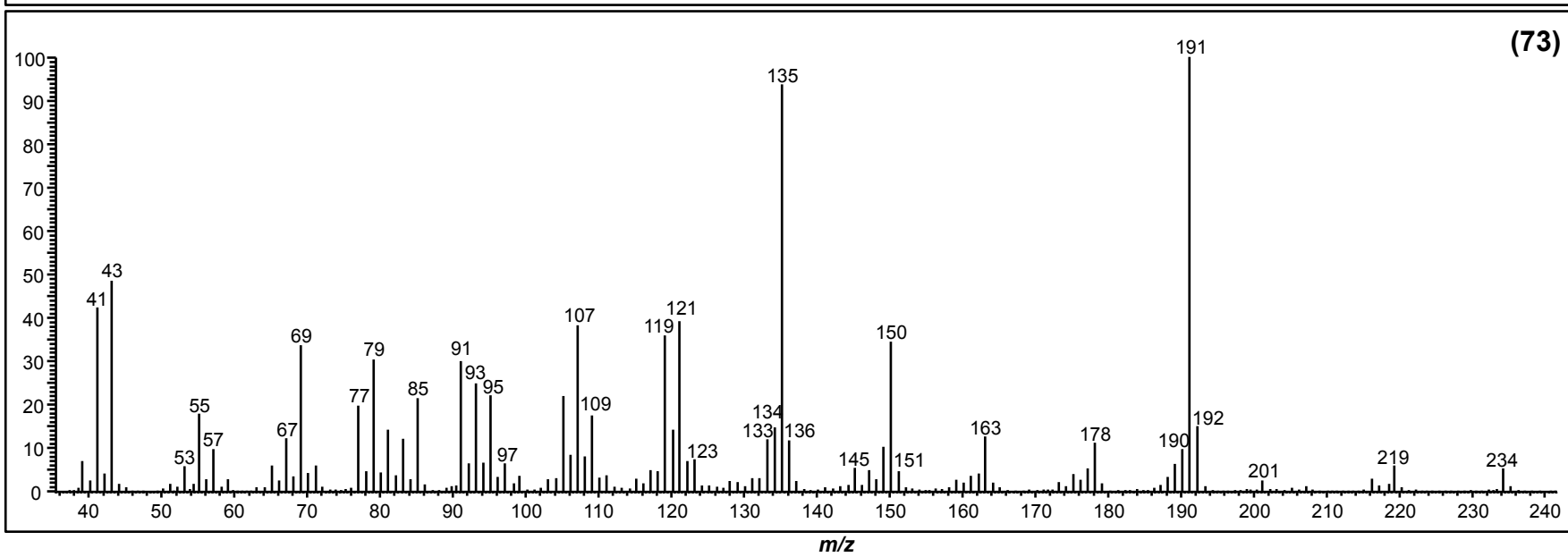

Supplement: Supplementary file 3 — (PDF 81 kb) [file 10886_2016_807_MOESM3_ESM.pdf]

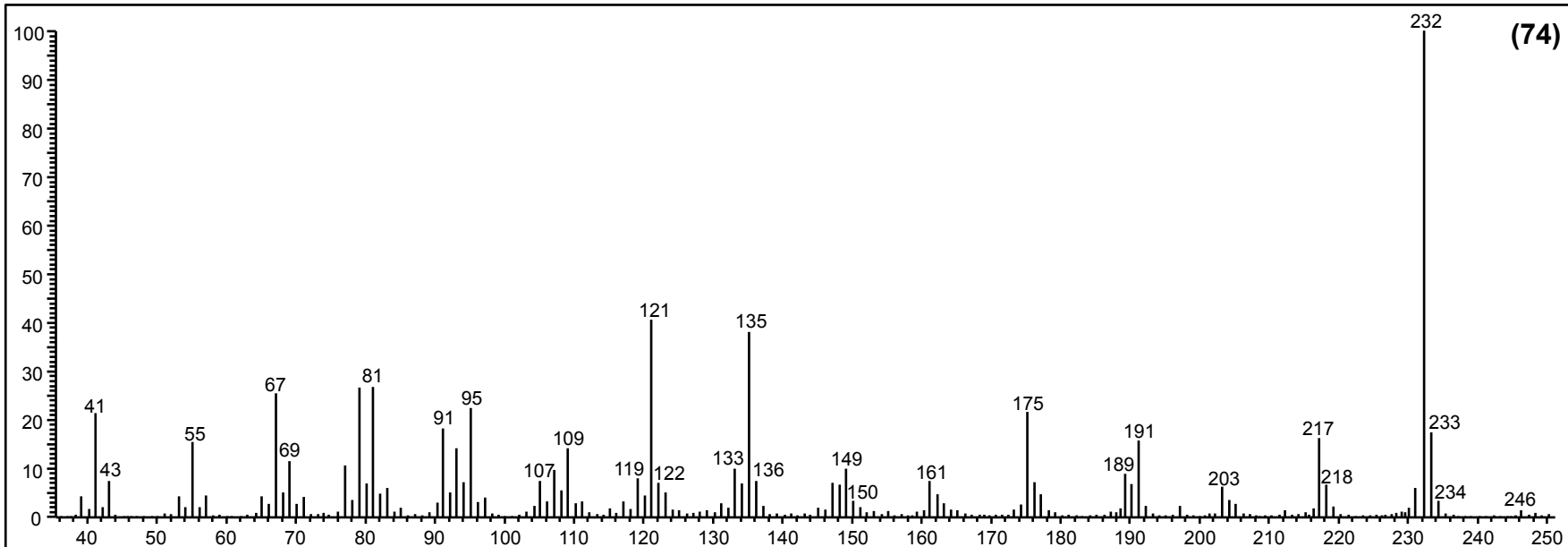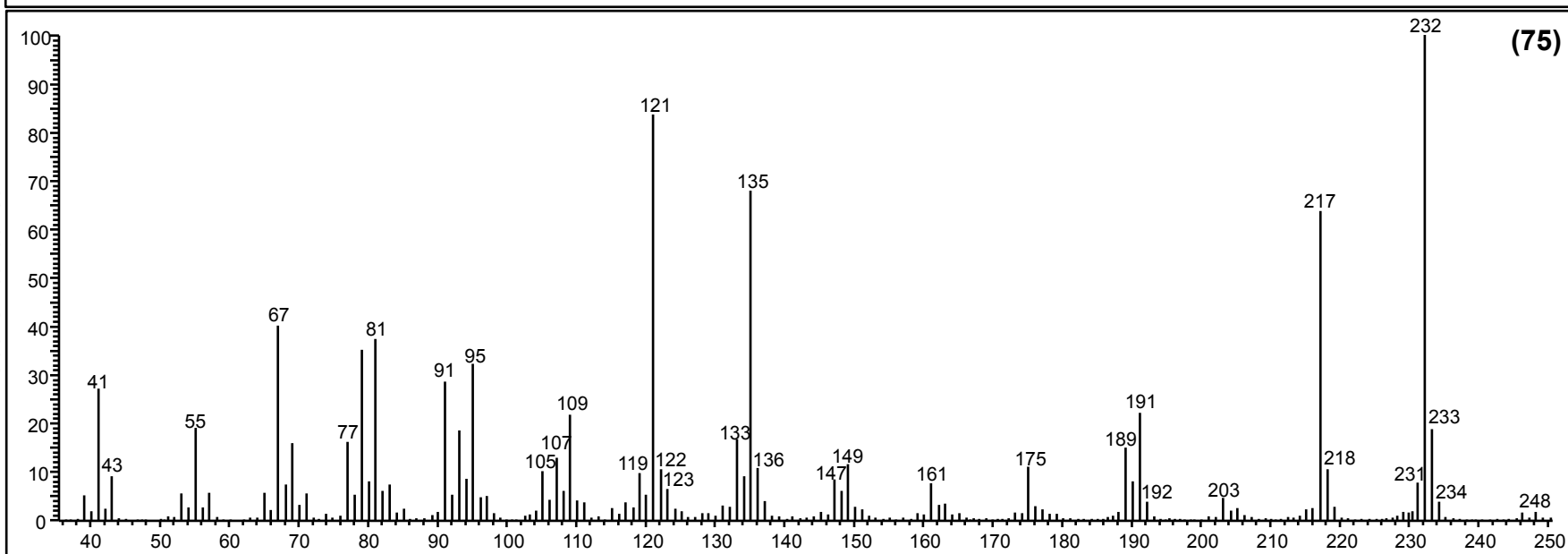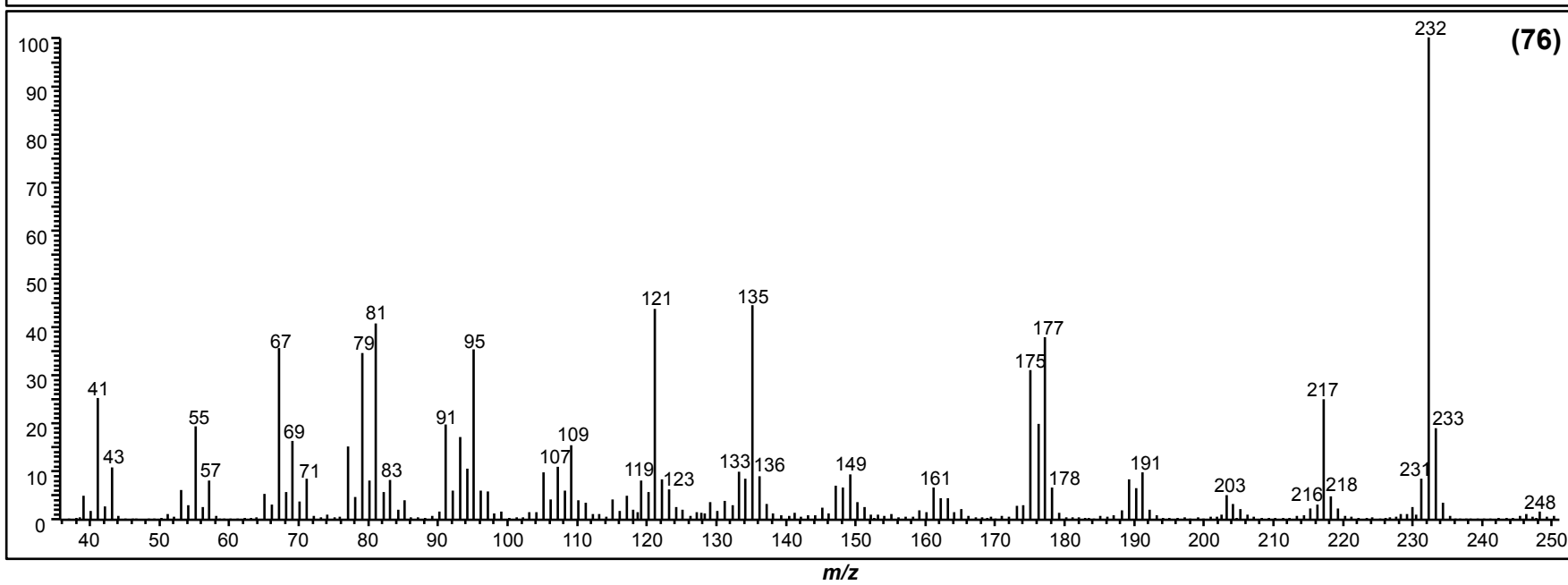

Supplement: Supplementary file 4 — (PDF 65 kb) [file 10886_2016_807_MOESM4_ESM.pdf]

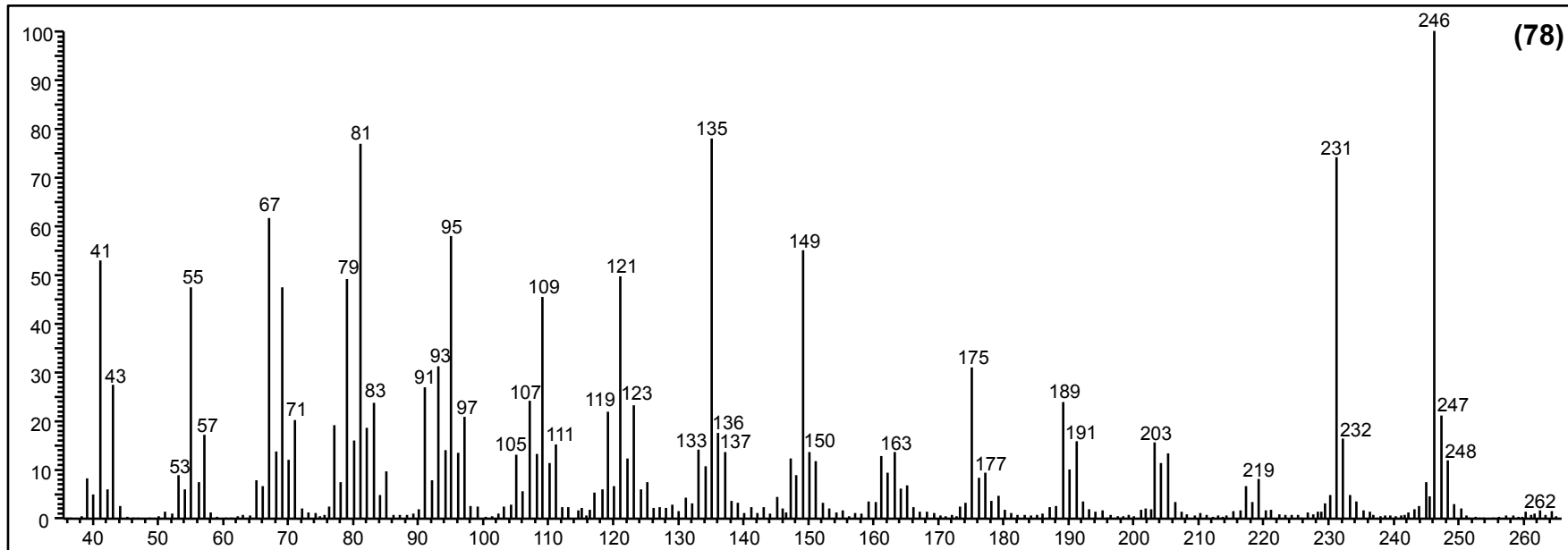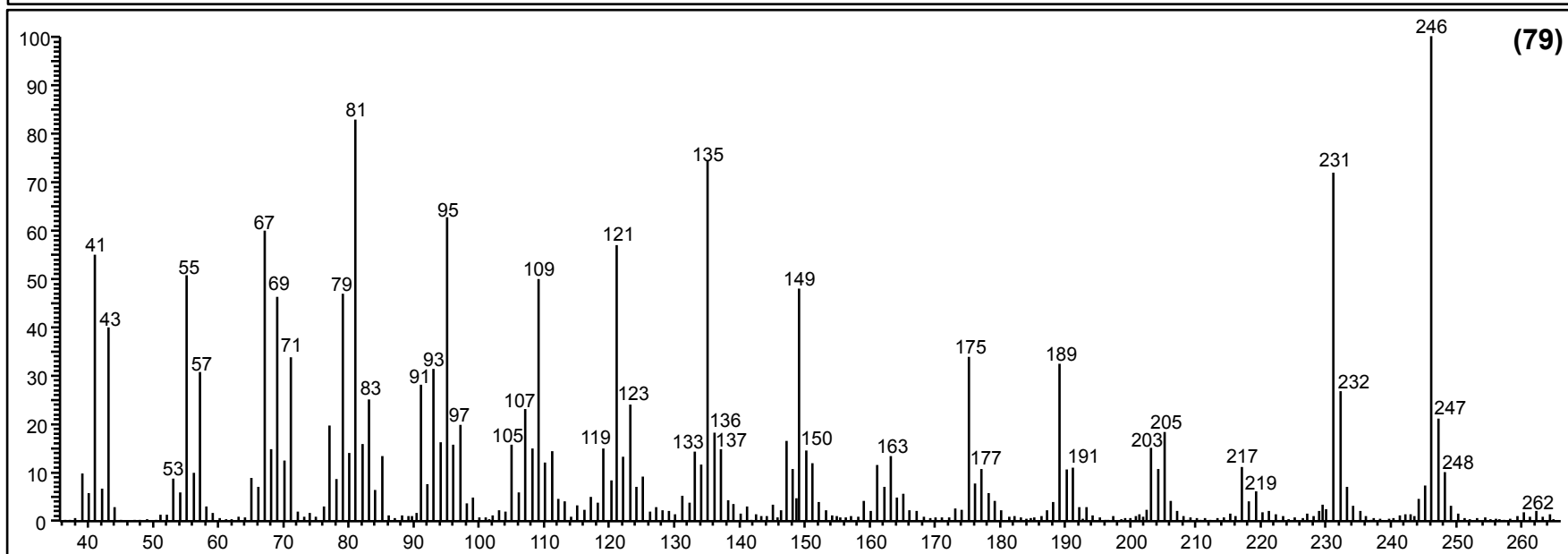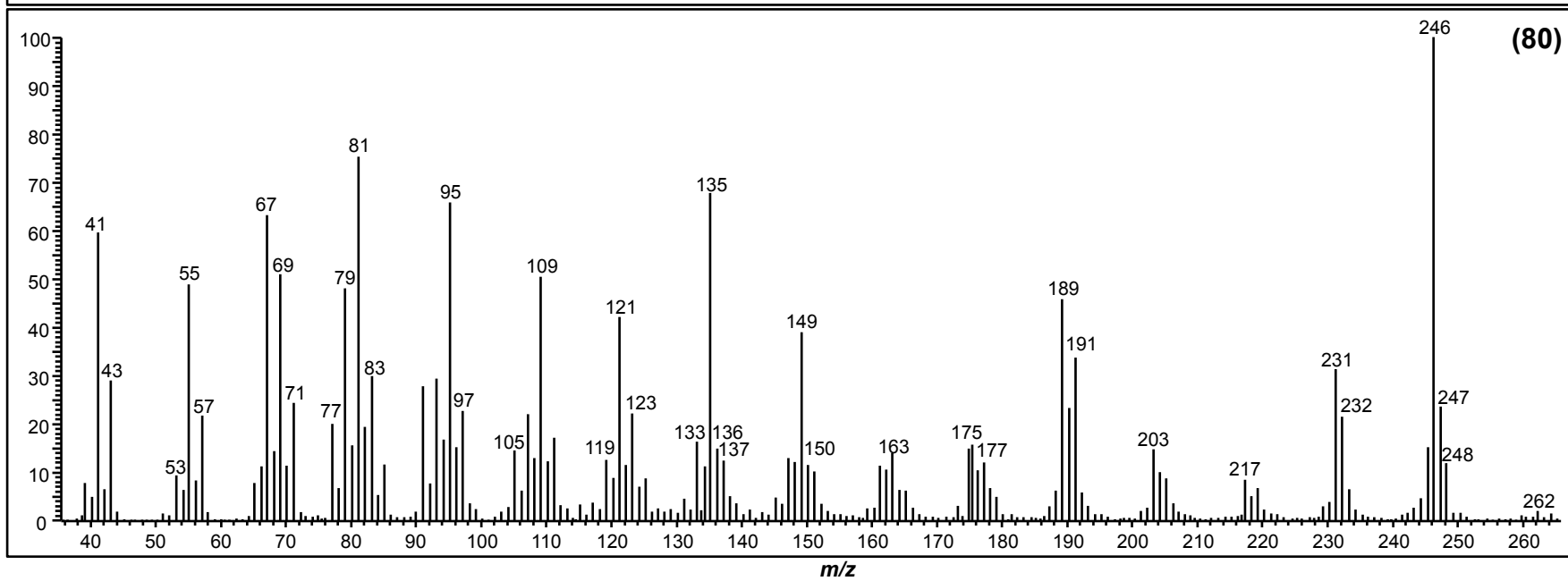

Supplement: Supplementary file 5 — (PDF 67 kb) [file 10886_2016_807_MOESM5_ESM.pdf]
